# Prognostic Value of Hepatitis B Virus Infection in Very Young Patients With Curatively Resected Breast Cancer: Analyses From an Endemic Area in China

**DOI:** 10.3389/fonc.2020.01403

**Published:** 2020-08-07

**Authors:** Ning Li, Qing-Qi Zhong, Xian-Rong Yang, Qi-Cai Wang, Di-Tian Zhang, Shaoquan Zheng, Lu Yang, Wei-Dong Wei

**Affiliations:** ^1^State Key Laboratory of Oncology in South China, Department of Breast Oncology, Sun Yat-sen University Cancer Center, Collaborative Innovation Center for Cancer Medicine, Guangzhou, China; ^2^Department of Thyroid and Breast Surgery, Longyan First Affiliated Hospital of Fujian Medical University, Longyan, China

**Keywords:** HBV, young breast cancer, prognosis, nomogram, survival

## Abstract

**Background:** Hepatitis B virus (HBV) infection has been associated with the risk and prognosis of many malignancies. Nevertheless, the association between HBV and the prognosis of breast cancer is unclear. The objectives of this study were to investigate the prognostic role of hepatitis B surface antigen (HBsAg) and to integrate HBsAg to establish nomograms for better prognostic prediction of very young patients with breast cancer.

**Methods:** This analysis was performed retrospectively in a cohort of 1,012 consecutive very young (≤35 at diagnosis) patients who received curative resection for breast cancer. The significance of HBsAg in the prognosis of these patients was investigated. Univariate and multivariate analyses were used to identify independent variables for disease-free survival (DFS) and overall survival (OS). Nomograms were built based on those identified variables.

**Results:** Overall, 140 of the 1,012 patients (13.8%) were seropositive for HBsAg. The median follow-up was 67.9 (95% CI, 64.4–71.4) months for the entire population. The HBsAg-positive cohort had significantly inferior DFS (HR, 1.66; 95% CI, 1.07–2.56; *P* = 0.021) and OS (HR, 1.75; 95% CI, 1.10–2.79; *P* = 0.016) as compared with the HBsAg-negative cohort. The rates of 10-year DFS and OS were 77.4 and 73.0% in the HBsAg-positive group and 84.1 and 85.6% in the HBsAg-negative group, respectively. In multivariable analysis, HBsAg status was identified as an independent significant unfavorable prognostic factor for DFS (*P* = 0.01) and OS (*P* = 0.04) in very young patients with breast cancer. Nomograms were established and displayed good calibration and acceptable discrimination. The C-index values for DFS and OS were 0.656 (95% CI: 0.620–0.691) and 0.738 (95% CI: 0.697–0.779), respectively. Based on the total prognostic scores (TPS) of the nomograms, 3 different prognosis groups were identified for DFS and OS.

**Conclusions:** HBsAg is an independent unfavorable prognostic factor for DFS and OS in very young patients with curatively resected breast cancer, and nomograms integrating HBsAg provide individual survival prediction to benefit prognosis evaluation and individualized therapy.

## Introduction

Globally, breast cancer is the most common cancer and the leading cause of cancer death for women, accounting for 24.2% of total cancer cases and 15.0% of total cancer deaths (1). Breast cancer has also been the top one malignancy in terms of incidence in Chinese women, constituting 12.2% of newly diagnosed cases and 9.6% of all deaths from breast cancer in the world (2). Although breast cancer occurs at a lower incidence in Chinese women than in western women, this disease occurs at a younger age in China than in high-income countries and China's contribution to global breast cancer rate is increasing rapidly (2). The disparities between young and old breast cancer include a higher mortality rate, higher risk of recurrence, poorer treatment response, and more aggressive phenotypes (3–5). Therefore, understanding the etiology and identifying novel prognostic factors are essential for early diagnosis, prognosis evaluation, early intervention, and personalized therapy in young patients with breast cancer.

Hepatitis B virus (HBV) infection is a serious public health dilemma, with ~350 million chronic carriers worldwide (6). China account for about a third of infection-associated cancer globally, driven by high prevalence of HBV and H pylori infection (7). Although China has made tremendous efforts in controlling HBV over the past 20 years and the prevalence of HBV in infants and children has remarkably declined (8), the hepatitis B surface antigen (HBsAg) prevalence is still high in Chinese adults, ranging from 6 to 9.5% (9–11). HBV is the leading cause of hepatocellular carcinoma and cholangiocarcinoma (12, 13). In addition, there is also accumulating evidence that HBV infection is associated with many extrahepatic malignancies (14), including non-Hodgkin's lymphoma (15), pancreatic cancer (16), gastric cancer (17), nasopharyngeal carcinoma (18), lung cancer (19), esophageal cancer (20), and ovarian cancer (21). Thus, it seems reasonable that HBV is an important factor in the development of extrahepatic malignancies in endemic areas.

Despite the facts that HBsAg status is one of the routine examinations in patients with operable breast cancer and several studies have showed that HBV is not associated with the risk of breast cancer (22, 23), the impact of HBV on the clinicopathological characteristics and prognosis of very young patients with breast cancer remains to be determined. Given that both early breast cancer and HBV are endemic in China, it is possible that HBV infection is associated with the prognosis of early breast cancer, even though the precise mechanisms are yet to be determined. It is crucial to address this issue since HBV has been reported to be found in breast cancer tissue (24). We therefore performed this study to investigate the HBsAg prevalence in very young breast cancer and the impact of HBsAg on the survival of these patients, and to establish nomograms to better predict prognosis for very young patients with breast cancer.

## Materials and Methods

### Patient Selection

A retrospective review was conducted in a cohort of 1,012 consecutive breast tumor women who were aged ≤35 years old and received curative resection for breast cancer at Sun Yat-sen University Cancer Center between May 1, 1999 and July 31, 2018. This study was conducted according to the ethical standards of the Declaration of Helsinki. Institutional Review Board approval was obtained from the Medical Ethics Committee of this cancer center. All patients were restaged by the eighth international classification system for breast cancer (25). Due to the retrospective nature of this study, informed consent was waived.

Information was collected from electronic patient records, and survival data were obtained from the follow-up registry of this center. The information collected included HBsAg status, laterality, type of breast surgery, type of axillary surgery, histological type, tumor grade, tumor-node-metastasis (TNM) stage, dates of surgery/relapse/death, status of estrogen receptor (ER), progesterone receptor (PR), human epidermal growth factor receptor 2 (HER-2), and Ki67. Breast cancers were classified as luminal A-like (ER+, PR≥20%+, HER2– and Ki67 <15%), luminal B-like (ER+ and/or PR+, HER2+/–), HER2-enriched (ER–, PR–, HER2+), or triple-negative (ER–, PR–, HER2–) subtypes.

Potentially eligible patients had to have curatively resected breast cancer without previous therapy other than neoadjuvant therapy, be aged 35 years old or below, and have definite information of HBsAg. The main exclusion criteria included benign tumor, not having surgery, having incomplete resection, previous malignant disease, hepatitis viral infections other than HBV, men patients, and insufficient data of survival or HBsAg.

### Statistical Analysis

The main objectives of this study were to compare disease-free survival (DFS) and overall survival (OS) between HBsAg-positive patients and HBsAg-negative patients. DFS was defined as the interval from the date of being diagnosed to the date of disease recurrence/metastasis or death from any cause. OS was defined as the interval from the date of being diagnosed to the date of death from any cause. Median follow-up was estimated by Kaplan-Meier analysis with reversed meaning of status indicator (26).

DFS and OS were estimated by the Kaplan–Meier method, and differences were compared by the log-rank test. Univariate and multivariate analyses with a Cox proportional hazards model were used to test for independent variables for DFS and OS. Covariates included laterality (left vs. right), type of surgery (breast-conserving surgery vs. mastectomy), type of axillary surgery (sentinel lymph node dissection vs. axillary lymph node dissection), histological type (ductal vs. others), tumor grade (grade III vs. grade I/II), T stage (T3/4 vs. T1/2), N stage (N2/3 vs. N1 = 0/1), HER2 status (positive vs. negative), molecular subtypes (triple-negative/HER2-enriched vs. luminal). All variables reaching a significance of 0.1 in univariate analyses were included in multivariate analysis.

The nomograms for predicting 3-, 5-, and 10-year DFS and OS were formulated based on the results of multivariate analysis by the “rms” package of R. The discrimination of the nomogram models was estimated by the Harrell's concordance index (C-index). The value of the C-index ranges from 0.5 to 1.0, with 0.5 implying a random chance and 1.0 indicating a perfect prediction. Calibration curves of the nomogram models for DFS, OS were plotted to assess the predictive value of the model (27). In addition, patients were divided into three different risk groups (high, intermediate, low) according to total prognostic scores (TPS). The total prognostic scores of patients were transformed into categorical variables based on cutoff points, which were determined by the minimum *P*-value from log-rank ×2 statistics with the X-tile program (28).

Pearson's chi-square test was used to compare categorical data. All *P*-values were two-sided, and *P* < 0.05 was considered statistically significant. Statistical analyses were performed by the SPSS software (SPSS Inc., version 16.0, Chicago, IL, USA) and R for Windows (version 3.6.2, http://www.r-project.org/).

### Data Availability

The authenticity of this article has been validated by uploading the key raw data onto the Research Data Deposit public platform (www.researchdata.org.cn), with the approval RDD number as RDDA2020001410. The data that support the findings of the study are available from the corresponding authors upon reasonable request.

## Results

### Patient Characteristics

**Figure 1:**
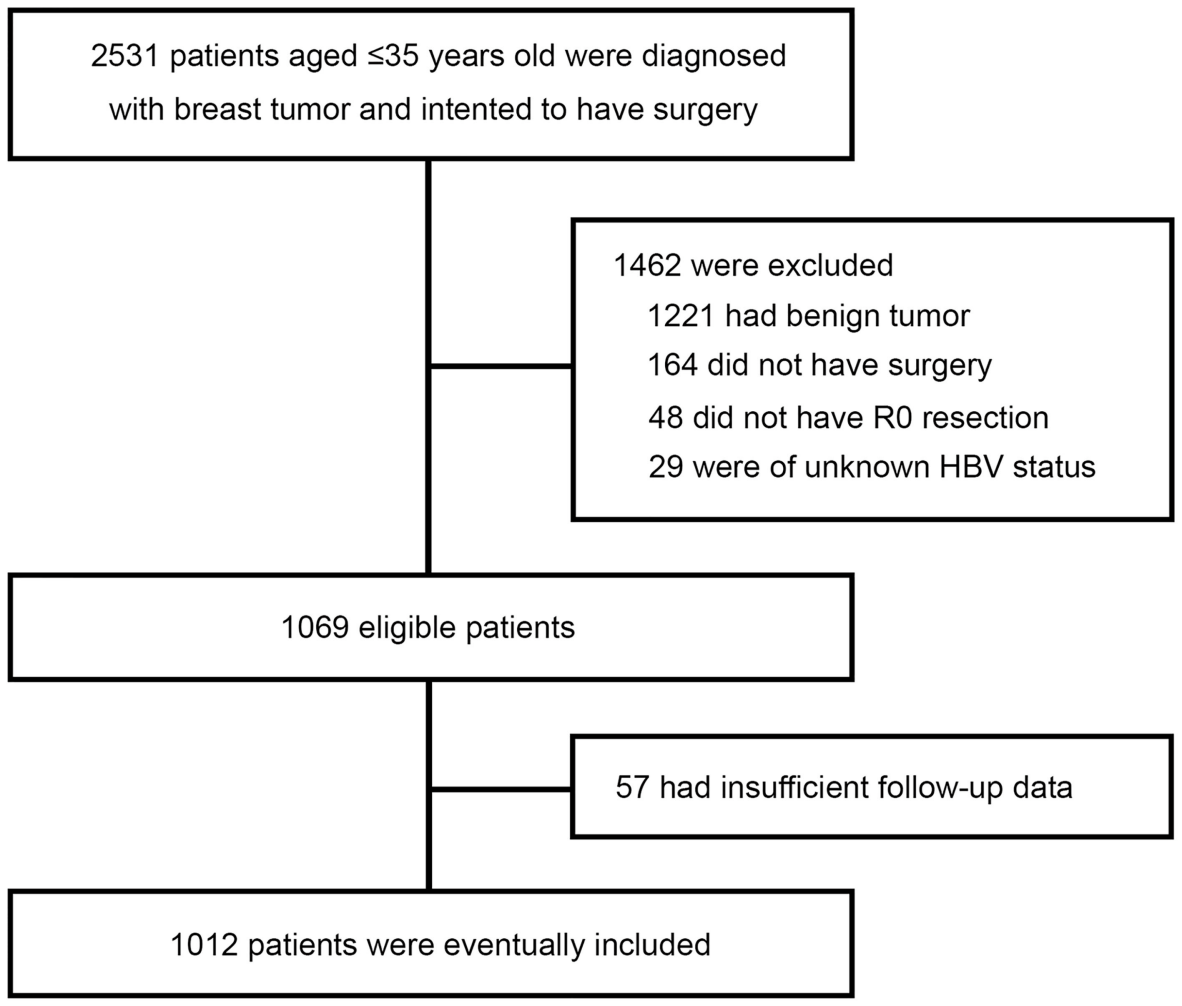
The process of patient selection.

A total of 2,531 very young (≤35 at diagnosis) breast tumor patients were screened, of whom 1,462 were excluded because of benign tumor (*n* = 1,221), not having surgery (*n* = 164), not having R0 resection (*n* = 48), or unknown HBsAg status (*n* = 29). With 57 further exclusions for insufficient follow-up data, a total of 1,012 patients with curative resection for breast cancer and aged 35 years or below were included in this study (Figure 1). The patients' characteristics are shown in Table 1. Overall, 140 of the 1,012 patients (13.8%) were seropositive for HBsAg. HBsAg-positive group and HBsAg-negative group were well-matched for basic characteristics, including laterality, type of breast and axillary surgery, histological type, tumor grade, T stage, N stage, and molecular subtypes. About 7 in 10 patients received mastectomy and most patients received axillary lymph node dissection (ALND).

**Table 1:** Patient characteristics by HBsAg status.

**Characteristics**	**HBsAg-positive (*N* = 140) No. (%)**	**HBsAg-negative (*N* = 872) No. (%)**	***P*-value**
**Laterality**			
Left	78 (55.7)	439 (50.3)	0.48
Right	61 (43.6)	428 (49.1)	
Bilateral	1 (0.7)	5 (0.6)	
**Type of breast surgery**			
Mastectomy	97 (69.3)	628 (72.0)	0.51
Breast-conserving surgery	43 (30.7)	244 (28.0)	
**Type of axillary surgery**			
ALND	115 (82.1)	738 (84.6)	0.45
SLND	25 (17.9)	134 (15.4)	
**Histological type**			
Ductal	129 (92.1)	792 (90.8)	0.88
Invasive lobular	1 (0.7)	8 (0.9)	
Other	10 (7.1)	72 (8.3)	
**Tumor grade**			
I	4 (2.9)	24 (2.8)	0.64
II	62 (44.3)	381 (43.7)	
III	42 (30.0)	315 (36.1)	
Unknown	32 (22.9)	152 (17.4)	
**T Stage**			
T1	49 (35.0)	284 (32.6)	0.62
T2	75 (53.6)	488 (56.0)	
T3	12 (8.6)	65 (7.5)	
T4	4 (2.9)	35 (4.0)	
**N Stage**			
N0	74 (52.9)	425 (48.7)	0.17
N1	41 (29.3)	237 (27.2)	
N2	17 (12.1)	124 (14.2)	
N3	8 (5.7)	86 (9.9)	
**HER2 status**			
Positive	29 (20.7)	231 (26.5)	0.58
Negative	95 (67.9)	552 (63.3)	
Unknown	16 (11.4)	89 (10.2)	
**Molecular subtypes**			
Luminal A	18 (12.9)	119 (13.6)	0.74
Luminal B	82 (58.6)	476 (54.6)	
HER2-enriched	8 (5.7)	79 (9.1)	
Triple negative	20 (14.3)	127 (14.6)	
Unknown	12 (8.6)	71 (8.1)	

HBV, Hepatitis B Virus; ALND, axillary lymph node dissection; SLND, sentinel lymph node dissection;

*HER2, human epidermal growth factor receptor-2*.

### Associations Between the Status of HBsAg and Survival

**Figure 2:**
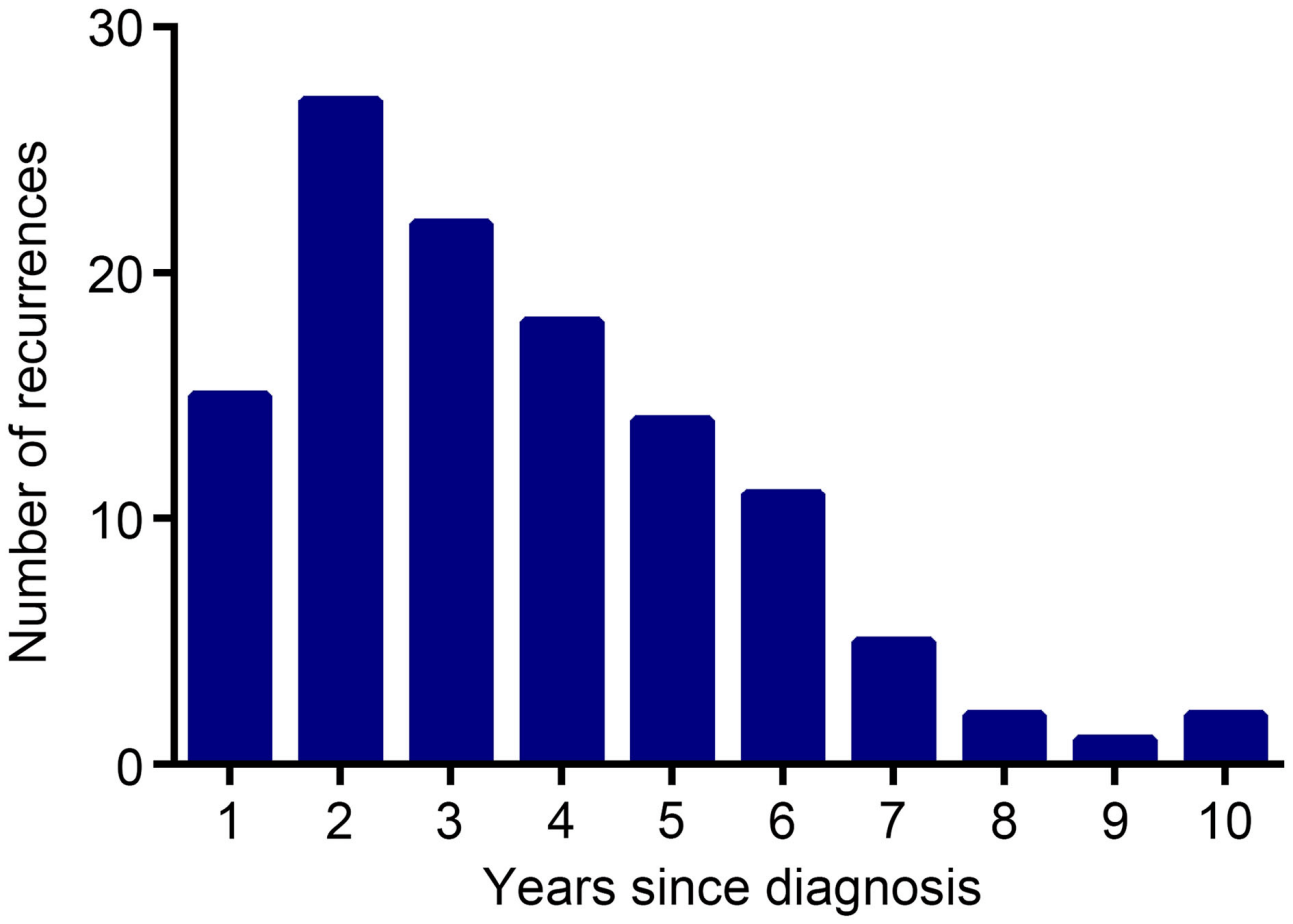
Number of recurrences by year of entire patients.

The median follow-up was 67.9 (95% CI, 64.4–71.4) months for the entire population. By the time of analysis (December 28, 2019), 122 instances of disease recurrence had occurred. The number of recurrences in each year of the follow-up is shown in Figure 2. Of note, although HBsAg-positive patients had a higher frequency of extrahepatic metastasis (15 vs. 9.1%, *P* = 0.029) than HBsAg-negative patients, they had a comparable frequency of liver metastasis (0.7 vs. 1.8%, *P* = 0.546) when compared with HBsAg-negative patients.

**Figure 3:**
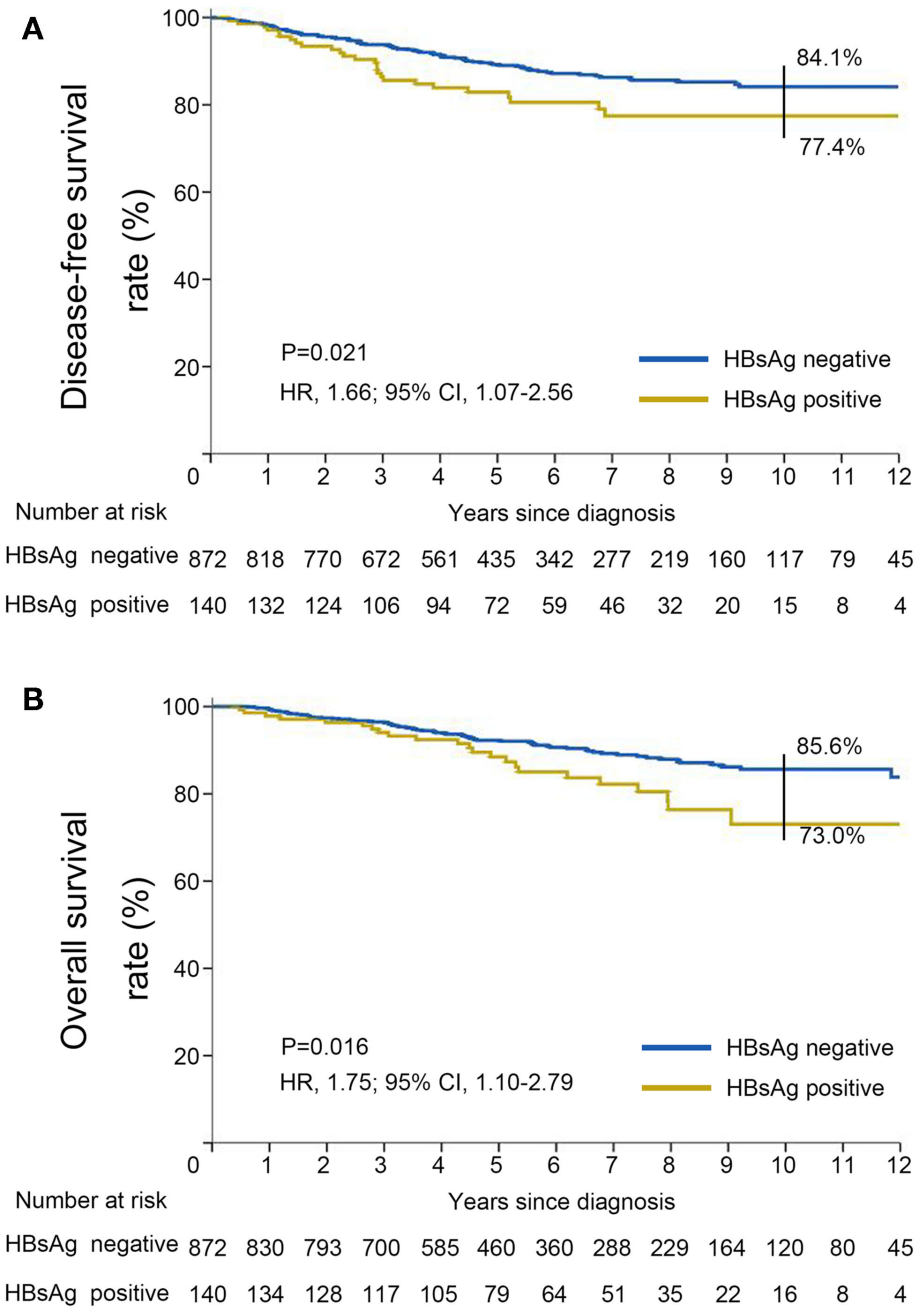
Kaplan–Meier curves for **(A)** disease-free survival and **(B)** overall survival stratified by HBsAg status in very young patients with breast cancer.

**Figure 4:**
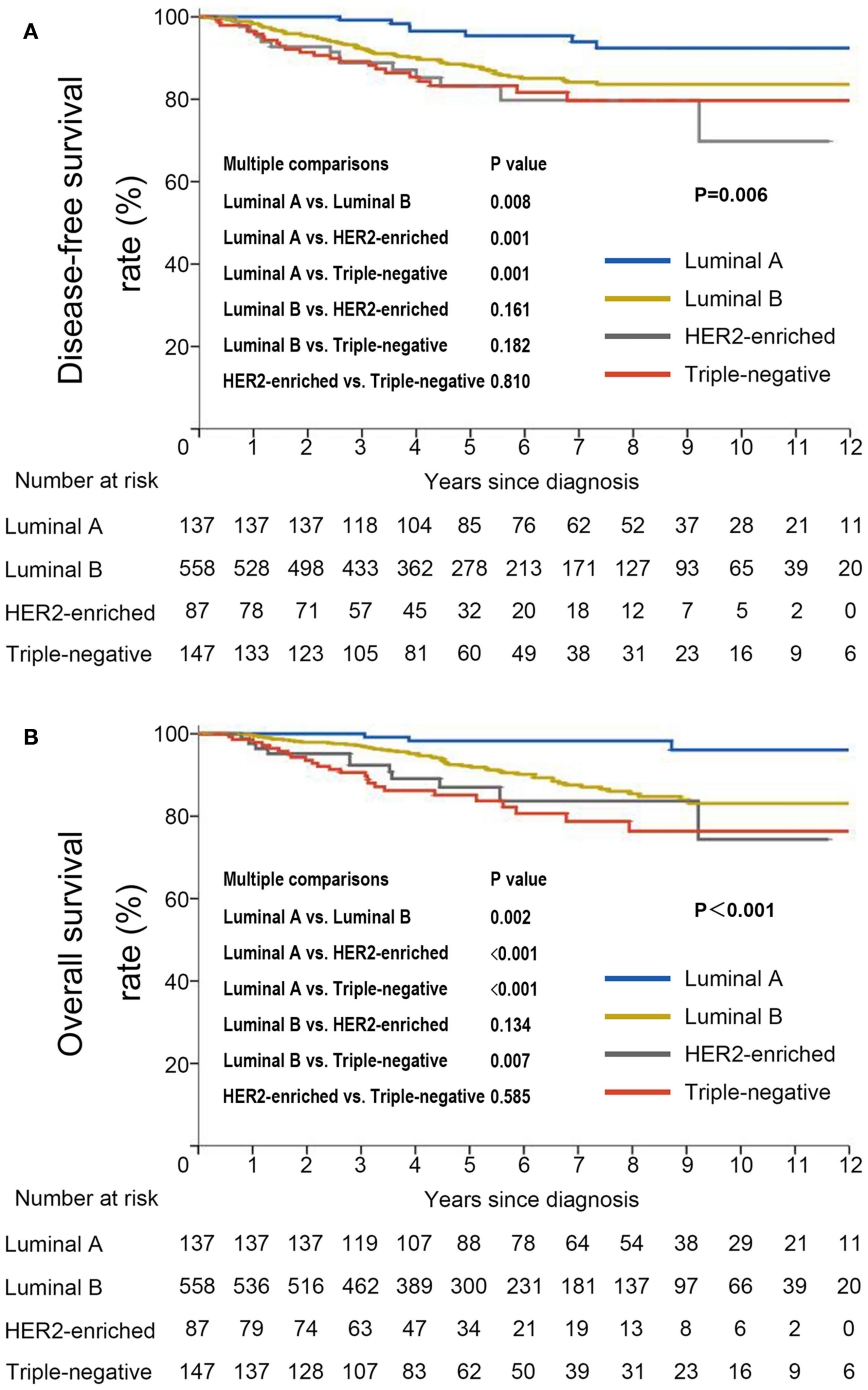
Kaplan–Meier curves for **(A)** disease-free survival and **(B)** overall survival stratified by molecular subtype in very young patients with breast cancer.

DFS was significantly shorter among those who were HBsAg-positive than among those who were HBsAg-negative (HR, 1.66; 95% CI, 1.07–2.56; *P* = 0.021). The rates of 10-year DFS were 77.4% in the HBsAg-positive group and 84.1% in the HBsAg-negative group, respectively (Figure 3A). A total of 101 death events had occurred by the data cutoff. HBsAg-positive group had significantly inferior OS compared with HBsAg-negative group (HR, 1.75; 95% CI, 1.10–2.79; *P* = 0.016), with a 10-year OS of 73.0 and 85.6%, respectively (Figure 3B). The association of HBsAg status and survival in each molecular subtype was further analyzed. As expected, DFS and OS were significantly longer among those in the luminal A subgroup, and the HER2-enriched and triple-negative groups had significantly shorter DFS and OS (Figures 4A,B). Notably, HBsAg-positive status was associated with shorter DFS (*P* = 0.027) and OS (*P* = 0.038) in the luminal B cohort. HBsAg-positive status was also associated with a slightly shorter DFS in the triple-negative cohort, and shorter OS in the HER2-enriched cohort, but statistical significance was not reached (Supplementary Figures 1, 2). There was no significant difference in DFS between HBsAg-positive patients and HBsAg-negative patients in luminal A and HER2-enriched cohorts, and in OS in luminal A and triple-negative cohorts (Supplementary Figures 1, 2).

**Figure 5:**
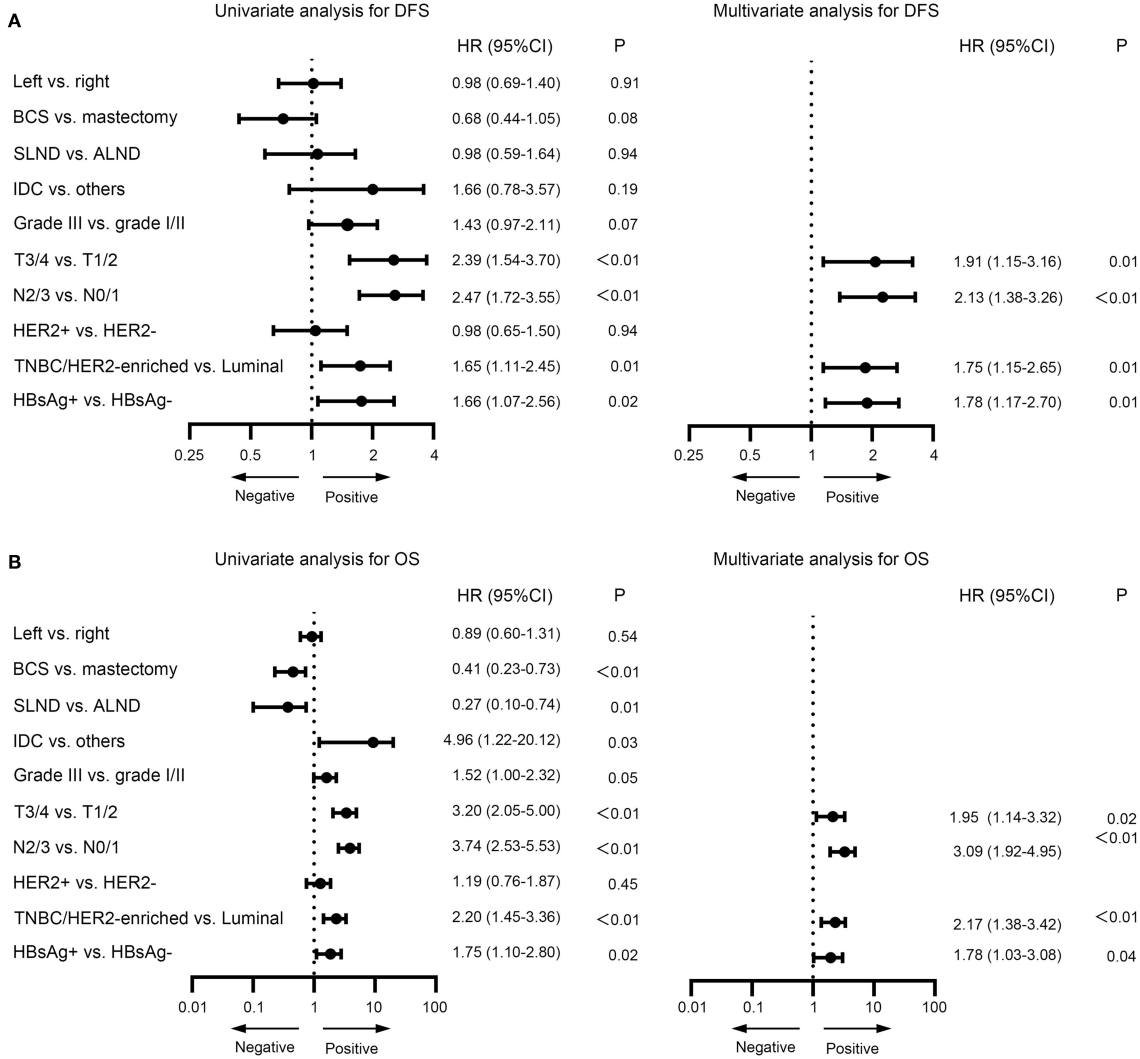
Univariate and multivariate analysis for disease-free survival **(A)** and overall survival **(B)** for very young patients with breast cancer.

In the univariate analysis, type of breast surgery, tumor grade, T stage, N stage, molecular subtype and HBsAg status were identified as significant prognostic factors for DFS (Figure 5A). When those variables were further analyzed in the multivariate analysis, we found that T stage (*P* = 0.01), N stage (*P* < 0.01), molecular subtype (*P* = 0.01) and HBsAg status (*P* = 0.01) remained statistically significant, indicating that they are significant, independent predictors for DFS (Figure 5A). By the same methods for OS, the results showed that T stage (*P* = 0.02), N stage (*P* < 0.01), molecular subtype (<0.01) and HBsAg status (*P* = 0.04) were independent prognostic factors for OS (Figure 5B). HBsAg-positive status is an independent negative prognostic factor for survival in very young breast cancer.

### Prognostic Nomograms For Very Young Breast Cancer Patients

**Figure 6:**
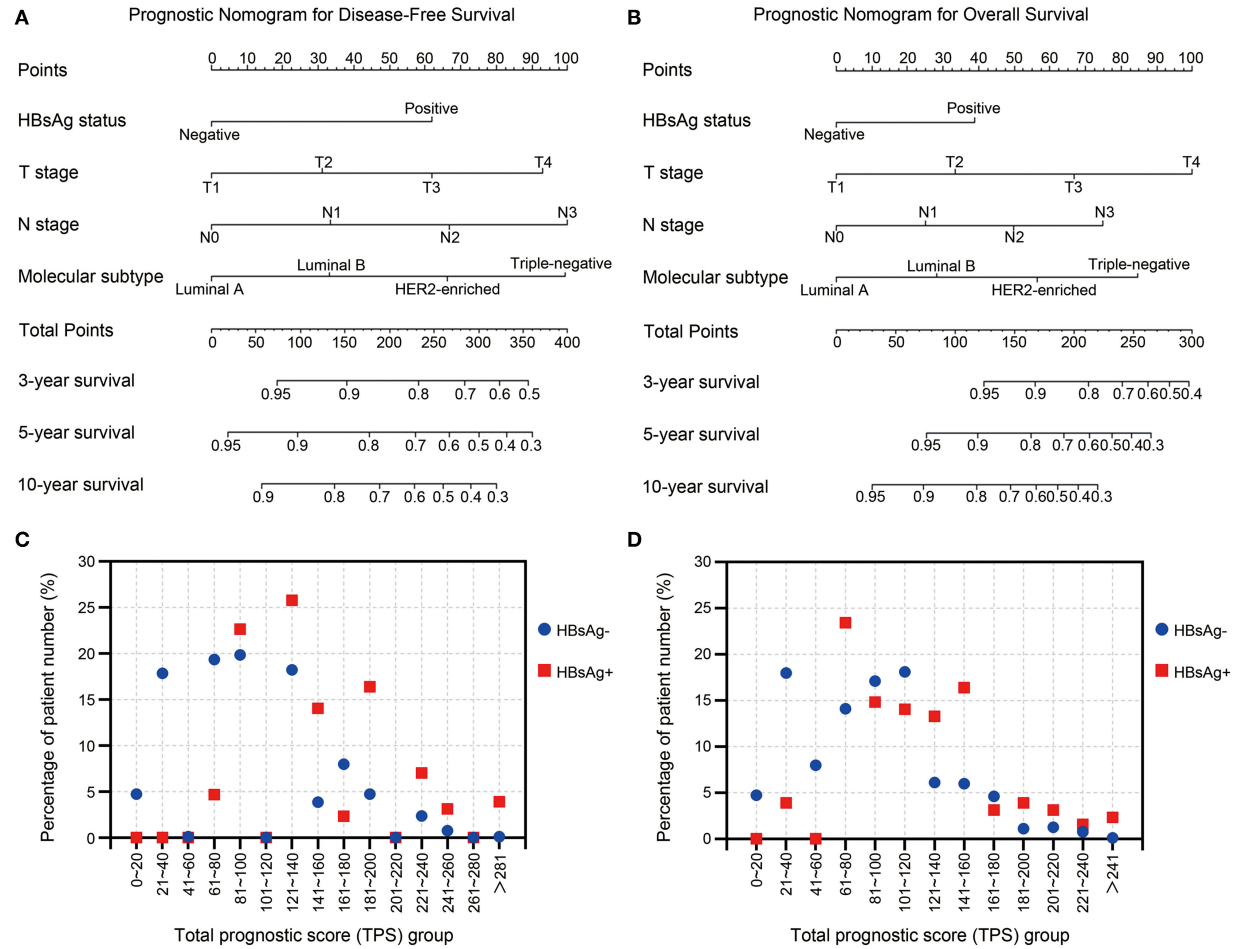
**(A,B)** Nomograms predicting 3-, 5-, and 10-year **(A)** disease-free survival and **(B)** overall survival for very young patients with breast cancer. **(C,D)** The scatter plots of percentage of patient number and groups of **(C)** total prognostic score for DFS and **(D)** total prognostic score for OS.

**Figure 7:**
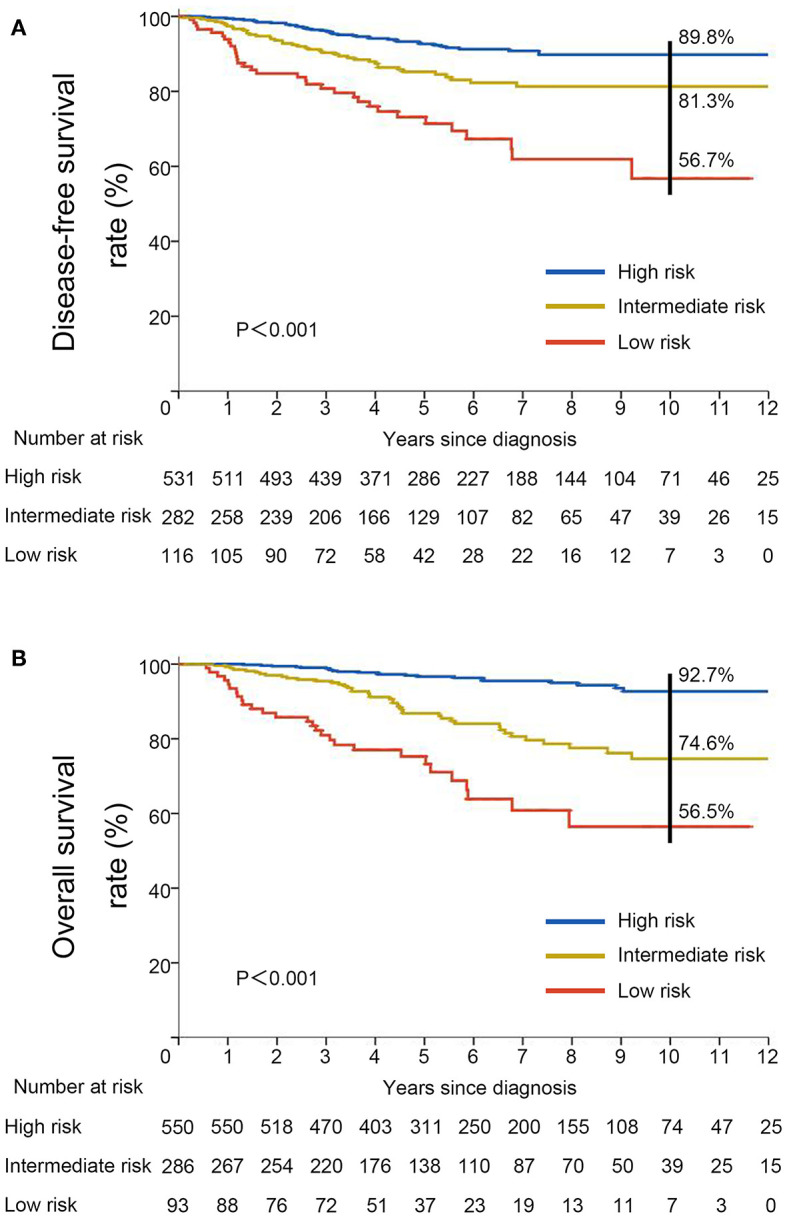
Kaplan–Meier curves for **(A)** disease-free survival and **(B)** overall survival stratified by risk groups based on total prognostic scores from nomogram models.

To better assess the DFS and OS of very young breast cancer patients, prognostic nomograms for DFS and OS were established, respectively. All the independent predictors of DFS and OS in the multivariate analysis were integrated into the nomogram models, and 3-, 5-, and 10-year survivals were graphically computed according to the characteristics of an individual patient (Figures 6A,B). The model's explanatory covariables consisted of HBsAg status, T stage, N stage, and molecular subtype. Patients with higher scores corresponded to inferior survival. The scatter plots for the TPS of DFS and OS, and percentage of patient number were presented in Figures 6C,D. The C-index values for DFS and OS were 0.656 (95% CI: 0.620–0.691) and 0.738 (95% CI: 0.697–0.779), respectively. The calibration curves for the probability of DFS and OS at 3, 5, or 10 year presented an optimal agreement between the prediction by nomogram and actual observation (Supplementary Figure 3). Next, we divided the patients into the following 3 groups based on the TPS of the nomogram model for DFS using the X-tile program: low-risk group (TPS, 0–100, 531 patients), intermediate-risk group (TPS, 101–163, 282 patients) and high-risk group (TPS >164, 116 patients). The 10-year DFS for low-risk group, intermediate-risk group, and high-risk group were 89.8, 81.3, and 56.7%, respectively. Survival analyses for DFS demonstrated significant discrimination between these three groups (*P* < 0.001, Figure 7A). Same procedures were performed for OS in the entire population, and patients were divided into the following 3 groups based on the TPS of the nomogram model for OS with the X-tile program: low-risk group (TPS, 0–100, 550 patients), intermediate-risk group (TPS, 101–154, 286 patients) and high-risk group (TPS >154, 93 patients). OS differences were also observed among three subgroups, with a 10-year OS of 92.7, 74.6, and 56.5% for low-risk, intermediate-risk and high-risk groups (*P* < 0.001, Figure 7B).

## Discussion

In this study we investigated the association of HBsAg status and very young breast cancer, and to our knowledge, report for the first time that HBsAg-positive status is associated with inferior DFS and OS from a population with a high prevalence of both HBV infection and young breast cancer. HBsAg status was identified as a significant unfavorable prognostic predictor for DFS and OS, independent of any other clinicopathological features of breast cancer, including T stage, N stage, and molecular subtype. We also integrated HBsAg to build nomograms to better predict prognosis for young patients with breast cancer. The results of our study demonstrated that the prevalence of HBsAg in young patients with breast cancer in southern China was 13.8%, which was in accordance with the 8–15% reported in the population of this endemic area (29). This result suggests that unlike cervical cancer (30), young breast cancer is not correlated with an increased prevalence of HBV infection. Indeed, breast cancer patients with HBsAg did not demonstrate a different pattern of characteristics. It is noteworthy that in our study, HBsAg did not increase the rate of liver metastases for very young patients with breast cancer. This results are comparable to those reported in previous studies for esophageal cancer and colorectal cancer (20, 31), which suggested that HBV infection is associated with decreased risk of liver metastasis in these malignancies.

Our study shows that HBsAg-positive status is associated with inferior DFS and OS in young patients with curatively resected breast cancer, decreasing the 10-year DFS and OS by 6.7 and 12.6%, respectively. The association between HBsAg-positive status and poor prognosis is in keeping with that demonstrated in nasopharyngeal carcinoma (32), lung cancer (19), and ovarian cancer (21). However, some studies regarding other cancers indicate that HBsAg-positive cancer patients had a favorable survival as compared with HBsAg-negative patients (20, 31). This discrepancy can be partly explained by the diversity and heterogeneity of different malignancies. The genetic or biological mechanisms underlying the inferior prognosis remain to be elucidated, but this may largely relate to the presence of hepatitis B X-interacting protein (HBXIP), which has been well-documented to function as an oncoprotein in breast cancer (33). HBXIP can act as a transactivator by activating certain genes including c-Myc, E2F1, STAT4, and Sp1 to play a crucial role in the progression of breast cancer (34). HBXIP is associated with controlling cell apoptosis and promoting cell proliferation by mTOC1 activation (35). HBXIP can also act as a modulation factor of cellular oxidative stress by competitively binding KEAP1 to enhance the progression of breast cancer (36). Previously studies showed that HBV is not associated with risk of breast cancer (22, 23). These results combined with the data of our study suggest that HBV is not a risk factor, but a prognostic factor for breast cancer.

Another reason for this might relate to the HBV reactivation in HBsAg-positive patients with breast cancer who were receiving chemotherapy. HBV reactivation occurs frequently in breast cancer patients who are HBV carriers while receiving cytotoxic chemotherapy (37). HBV reactivation can result in liver failure and interruption of the chemotherapy schedule. Other potential mechanisms underlying the association between the HBsAg and patient survival include the immune suppression. Chronic HBV infection is characterized by the failure to elicit an effective adaptive immune response and the immune modulation of key innate immune response (38). Chronic HBV infection can lead to immune anergy and impair the function of the immune system, which has long been deemed to protect the host against the development of non-viral cancers (39). These reasons together could partially explain the positive association between HBsAg-positive status and the poor prognosis in young patients with breast cancer.

In this study, we combined HBsAg status with clinicopathological characteristics to establish effective prognostic nomogram models of DFS and OS for very young patients with breast cancer. Both nomograms showed good calibration and acceptable discrimination. These nomogram models can be used for prognosis evaluation at diagnosis for very young patients with breast cancer, and may benefit patient counseling and personalized therapy for these patients. We adopted X-tile program to divide these patients into three risk groups based on TPS from nomograms for DFS and OS. The survival curves for DFS and OS separated very well. Thus, special attention should be paid to and active surveillance should be conducted over patients with high risk group for DFS and OS.

This study nevertheless has certain limitations that should be noted. First, this study was retrospective in nature and we cannot rule out the impact of selection bias. Second, the sample size was relatively small and the sample sizes of the two cohorts were unequal, as only 140 patients were HBsAg-positive. The small sample size may be insufficient to allow us to perform subgroup analysis by each molecular subtype. Another limitation is that Cantonese constitute most of our study population. The monotonicity of the study population confines the universality of our results. Furthermore, the information was insufficient to perform other analysis, such as that of hepatic function and HBV DNA copy number. Nevertheless, the results of our study provide what to our knowledge is the first evidence of the impact of HBsAg on the prognosis of very young patients with breast cancer.

In conclusion, we have demonstrated in a retrospective study that HBsAg is an independent unfavorable prognostic factor for patients with very young breast cancer. Further prospective studies involving varied ethnic populations are warranted to confirm the prognostic value of HBsAg status in very young breast cancer, and simultaneously other potential clinicopathologic factors for breast cancer and HBV infection are required to be taken into account. The mechanisms of the impact of HBV infection on the progression of breast cancer also need to be elucidated.

## Data Availability Statement

The datasets generated for this study are available on request to the corresponding author.

## Ethics Statement

The studies involving human participants were reviewed and approved by the Medical Ethics Committee of Sun Yat-sen University Cancer Center. Written informed consent for participation was not required for this study in accordance with the national legislation and the institutional requirements.

## Author Contributions

NL, Q-QZ, LY, and W-DW designed the study. NL, Q-QZ, X-RY, Q-CW, D-TZ, and SZ collected the data. NL, Q-QZ, LY, and W-DW interpreted and analyzed the data. All authors were involved in writing the article and approved the final version.

## Conflict of Interest

The authors declare that the research was conducted in the absence of any commercial or financial relationships that could be construed as a potential conflict of interest.
