# Supplementary material for: Prognostic Value of Hepatitis B Virus Infection in Very Young Patients With Curatively Resected Breast Cancer: Analyses From an Endemic Area in China
Source: Front Oncol. 2020 Aug 7;10:1403. doi: 10.3389/fonc.2020.01403 (PMC7426807; doi:10.3389/fonc.2020.01403)

**Supplementary Figure 1**. Kaplan–Meier curves of disease-free survival stratified by HBsAg status in very young patients with (A) luminal A, (B) luminal B, (C) HER2-enriched, and (D) triple-negative breast cancer.


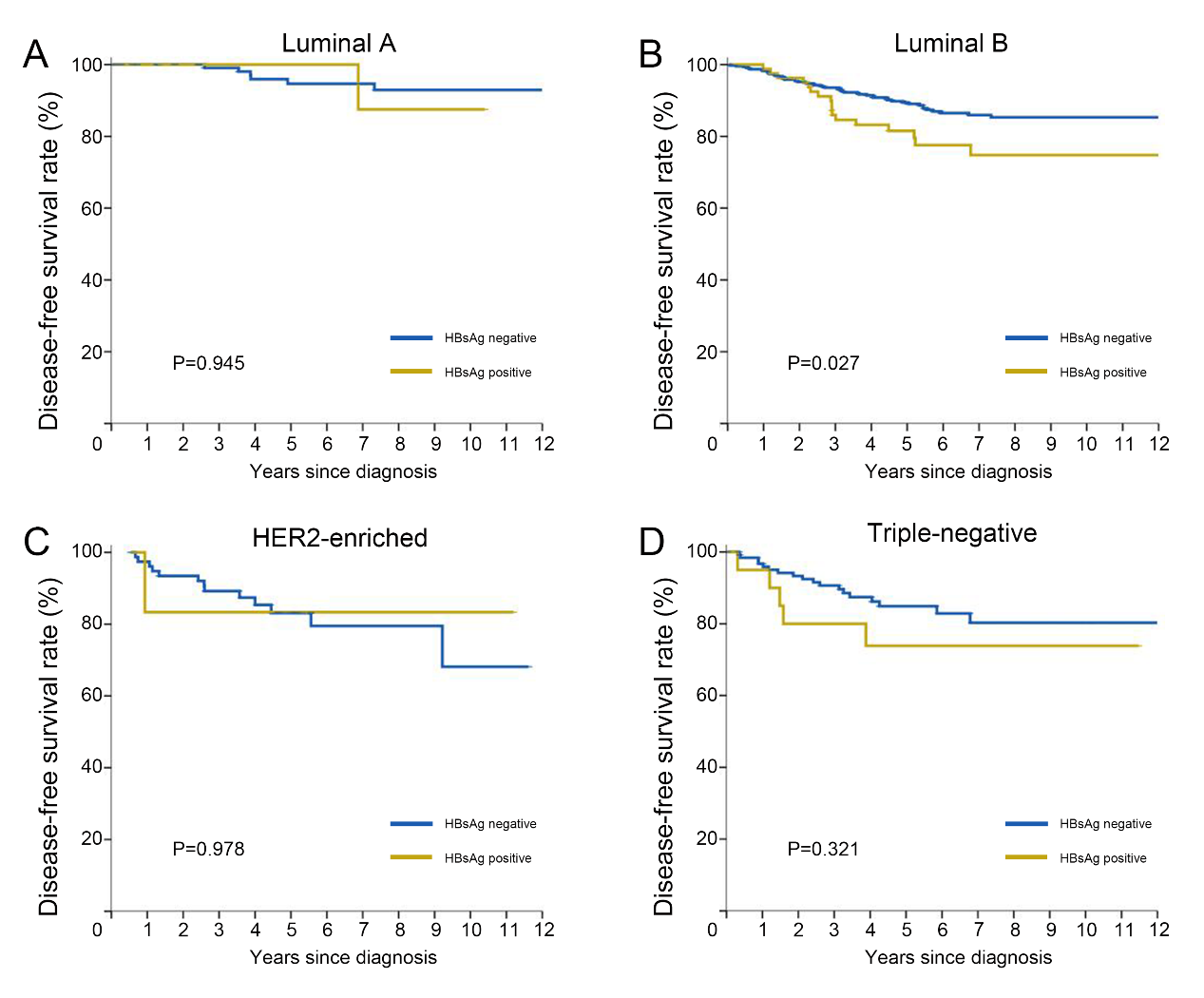


**Supplementary Figure 2**. Kaplan–Meier curves of overall survival stratified by HBsAg status in very young patients with (A) luminal A, (B) luminal B, (C) HER2-enriched, and (D) triple-negative breast cancer.


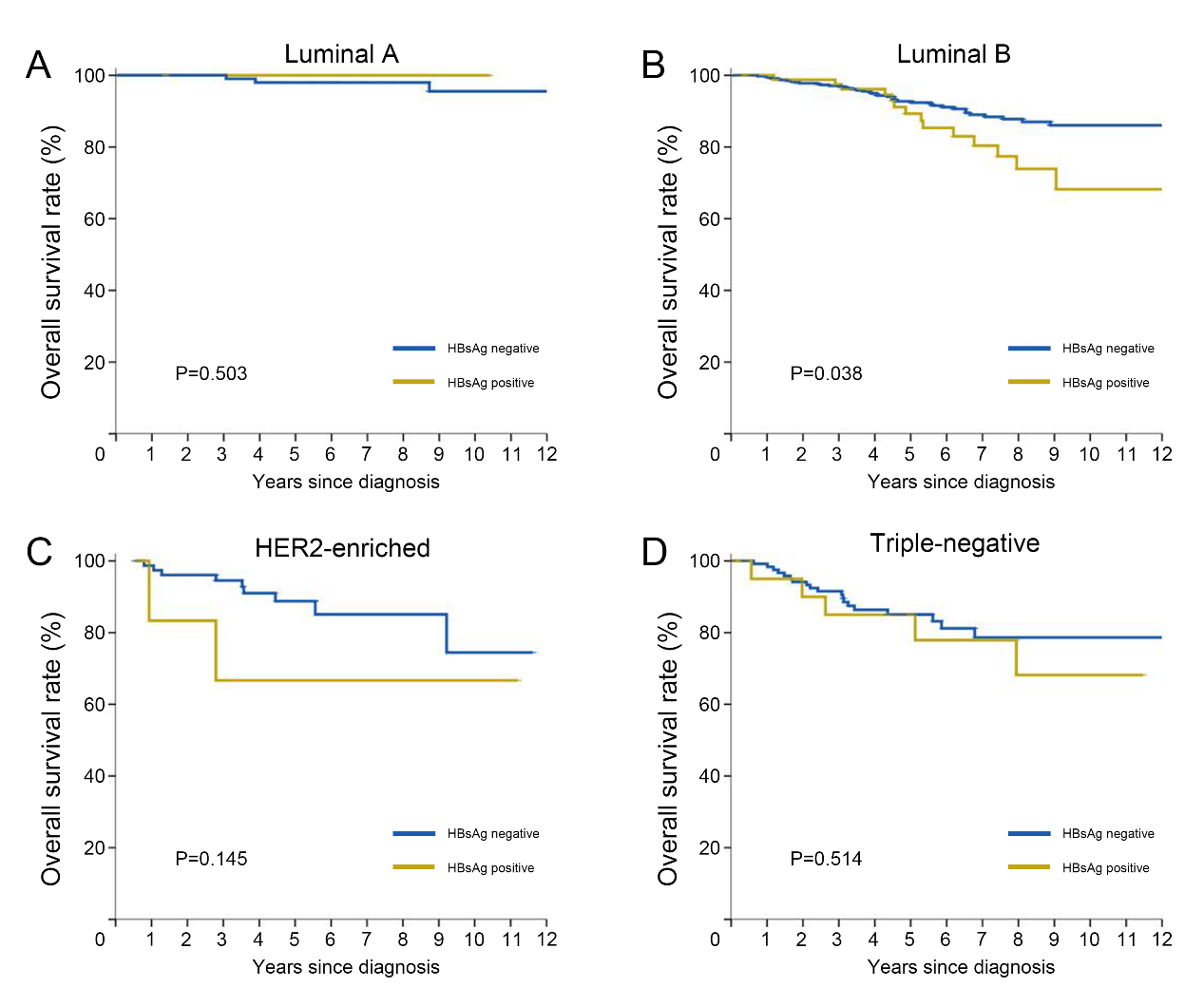


**Supplementary Figure 3**. The calibration curves for predicting disease-free survival at (A) 3 years, (B) 5 years and (C) 10 years, and overall survival at (D) 3 years, (E) 5 years and (F) 10 years in very young patients with breast cancer. Solid black line: performance of the nomogram model. The diagonal gray line represents a perfect estimation.


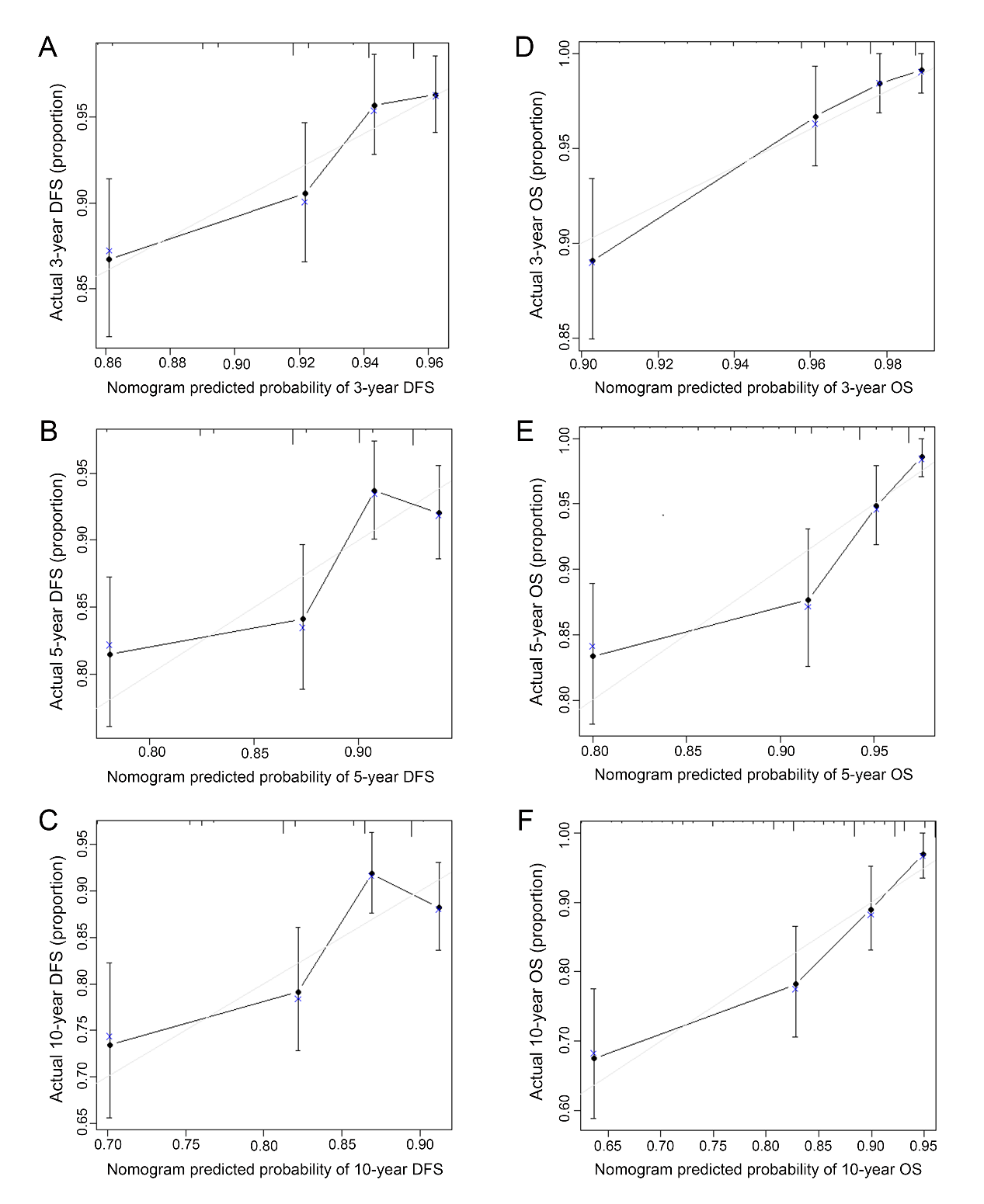

Supplement: Supplementary file 1 [file Data_Sheet_1.DOCX]
